# Unusual Pathology in a Kidney from a Heart-Transplant Patient

**DOI:** 10.1155/2017/1084718

**Published:** 2017-11-05

**Authors:** Marie Larcher, Audey Delas, Clément Delmas, Olivier Cointault, Camille Dambrin, Arnaud Del Bello, Nassim Kamar

**Affiliations:** ^1^Department of Nephrology and Organ Transplantation, CHU Rangueil, Toulouse, France; ^2^Department of Pathology, Institut du Cancer de Toulouse, Toulouse, France; ^3^Université Paul Sabatier, Toulouse, France; ^4^Department of Cardiology, CHU Rangueil, Toulouse, France; ^5^Department of Heart Surgery, CHU Rangueil, Toulouse, France; ^6^INSERM U1043, IFR–BMT, CHU Purpan, Toulouse, France

## Abstract

Acute kidney injury (AKI) is often observed after heart transplantation. In this setting, acute tubular necrosis is the main histological finding on kidneys. We report the unusual pathology found in a kidney from a heart-transplant patient. The patient experienced several hemodynamic insults, massive transfusion, and implantation of a mechanical circulatory-support device before heart transplantation: there was prolonged AKI after transplantation. A kidney biopsy revealed acute tubular necrosis and renal hemosiderosis, which was probably related to the transfusion and to mechanical circulatory-support device-induced intravascular hemolysis. Assessment of iron during resuscitation could have prevented, at least partly, AKI.

## 1. Background

Acute kidney injury (AKI) is often observed after heart transplantation and can be a predictor of a patient's survival. AKI is mainly associated with cardiopulmonary failure or low cardiac output before transplantation, with the hemodynamic effects from prolonged surgery and blood loss, or with the nephrotoxic effects of calcineurin inhibitors (CNIs) and sepsis [1]. When a kidney biopsy is performed in this setting, the usual histological findings on kidneys are acute tubular necrosis, hypertensive nephroangiosclerosis, and signs of CNIs toxicity [1]. Herein, we report an unusual pathological condition found in a kidney from a heart-transplant patient.

## 2. Case Report

A 57-year-old man underwent heart transplantation. Seven months earlier, he had been admitted for cardiogenic shock caused by a myocardial infarction. This had required peripheral venoarterial extracorporeal membrane oxygenation for 5 days, followed by implantation of a biventricular-assist device one week later. Over the next few days, the patient presented with four episodes of bloody pericardial effusion that required drainage; the last episode required open surgery.

Between the first admission and heart transplantation, the patient had presented with several episodes of infection and had remained in the intensive-care unit under mechanical ventilation.

During the 6-month follow-up after transplantation, the patient experienced no acute-rejection episodes while receiving an immunosuppressive regimen that combined tacrolimus (target trough level 5–8 ng/mL), mycophenolate (1 g/d), and low-dose steroids (5 mg/d). Left ventricular function was 50%.

**Figure 1:**
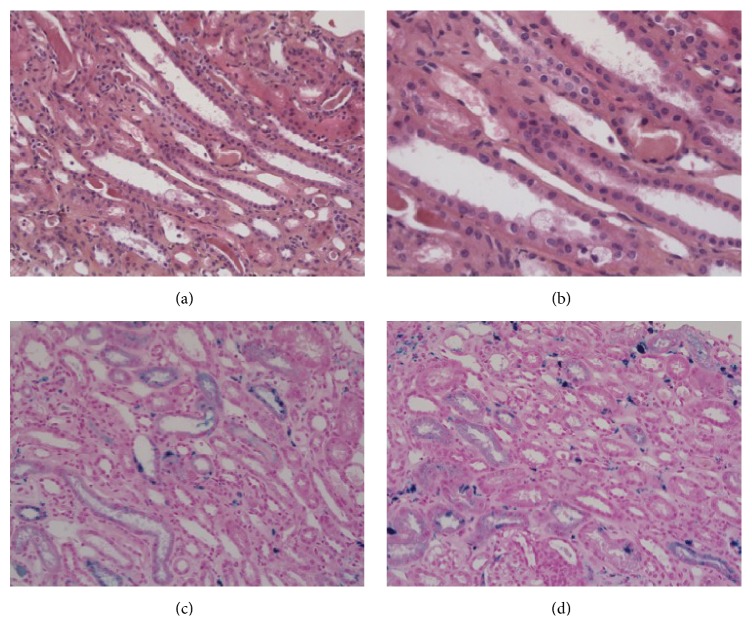
Kidney biopsy: (a) and (b): hematoxylin and eosin stain showing acute tubular necrosis ((a) ×20; (b) ×40). (c) and (d): kidney biopsy showing significant hemosiderin deposition, as highlighted by positive Perls staining ((c) and (d) ×20).

Before the myocardial infarction, the patient had impaired kidney function of undetermined origin. Blood parameters assessed 1 month before admission yielded the following results: serum creatinine level at 133 *μ*mol/L, CKD Epi estimated glomerular-filtration rate of 50 mL/min, proteinuria at 0.28 g/d, liver enzymes levels within the normal ranges, and hemoglobin level at 12.5 g/dL. After the cardiac shock, which occurred at initial admission, the patient became anuric, his serum creatinine level increased to 500 *μ*mol/L, and he required continuous venovenous hemodiafiltration until 20 days after heart transplantation. At that time, urine output had increased and so intermittent dialysis (three times per week) was started. At 2.5 months after transplantation, despite 2 L per day of diuresis, the patient had not recovered kidney function: thus a kidney biopsy was performed (Figure 1). This showed acute tubular necrosis and significant deposition of hemosiderin, as shown by positive Perls staining. However, staining for immunoglobulin and complement was negative. A biological work-up revealed iron overload, that is, ferritin of 7,000 ng/mL and transferrin saturation of 51%. Soluble transferrin receptor was decreased to 1.3 mg/L (*N*: 2.2–5). The* HFE C282Y* gene was not mutated.

Magnetic-resonance imaging of the liver showed iron overload, evaluated at 230 mg/mL. Perls staining of the explanted heart and a biopsy of the heart allograft were negative. Assessed retrospectively, the patient received 240 red blood cell units, that is, ~72 L of blood units, during the stay in hospital and during the bleeding episodes. In addition, before the heart transplant, the patient had experienced hemolysis induced by the biventricular-assist device; that is, lactate dehydrogenase levels were increased, haptoglobin levels were undetectable, and fragmented erythrocytes were observed on blood smears. Between the initial admission and transplantation, his hemoglobin level ranged between 7 and 10 g/dL despite massive transfusion and recombinant erythropoietin support.

At 3 months after heart transplantation, iron chelation was started. At 6 months after transplantation, ferritin level was decreased to 3,165 ng/mL and transferrin saturation was 81%. Soluble transferrin receptor was also decreased to 1.56 mg/L. Kidney function was slightly improved, and the patient only required one dialysis session per week instead of three. However, the patient died six months later from septic shock.

## 3. Discussion

AKI is frequently observed after heart transplantation and is mainly related to acute tubular necrosis. This was the case in our heart-transplant patient, who had suffered from several hemodynamic insults. However, surprisingly, pathological examination of the kidney biopsy revealed the presence of hemosiderin deposition that may have contributed to the acute tubular necrosis and AKI. Hemosiderin deposition in the kidney has been described in different types of chronic hemolytic anemia, such as autoimmune hemolytic anemia, paroxysmal nocturnal hemoglobinuria, and sickle-cell anemia [2–4], as well as hemochromatosis. Intravascular hemolysis can also occur after cardiac-valve replacement and residual valvular regurgitation or a perivalvular leak and is a well-known complication from mechanical circulatory-support devices [5].

During hemolysis episode, the hemoglobin alpha-beta dimers are released from the red blood cell. They are then filtered by the glomerulus and taken up by renal proximal tubular cells where they are degraded [4, 6]. The free chelated iron is stored as hemosiderin in the lysosomes. In case of hypovolemia, accumulation of hemosiderin can induce acute tubular necrosis and acute renal failure [4, 6]. Hypothesis for heme-induced nephrotoxicity is its lipophilic, oxidant, proinflammatory, and apoptotic properties. Mitochondria seem to be vulnerable to heme-mediated toxic effect [7].

In this reported case, the iron overload was caused by massive transfusions and intravascular hemolysis induced by the biventricular-assist device, which may have contributed to kidney failure. Hence, in patients that receive a massive transfusion and/or who have a mechanical circulatory-support device, iron levels should be assessed regularly and iron chelation should be proposed if necessary. According to the patient's medical history before transplantation, hemosiderin deposition should be suspected in those that have a heart transplant and present with prolonged AKI after transplantation.
